# AAL Platform with a “De Facto” Standard Communication Interface (TICO): Training in Home Control in Special Education

**DOI:** 10.3390/s17102320

**Published:** 2017-10-12

**Authors:** Miguel A. Guillomía San Bartolomé, Jorge L. Falcó Boudet, José Ignacio Artigas Maestre, Ana Sánchez Agustín

**Affiliations:** 1 Ingeniería Electrónica y Comunicaciones, Universidad de Zaragoza, María de Luna 1, 50018 Zaragoza, Spain; 601084@unizar.es (M.A.G.S.B.); jiartigas@unizar.es (J.I.A.M.); 2 Alborada Special Education School, Andador Pilar Cuartero sn, 50018 Zaragoza, Spain; asanchezag@educa.aragon.es

**Keywords:** Ambient Assisted Living (AAL), accessible interfaces, special education, assistive technology, collaborative design, personal autonomy, services provision, human/system interactions

## Abstract

Framed within a long-term cooperation between university and special education teachers, training in alternative communication skills and home control was realized using the “TICO” interface, a communication panel editor extensively used in special education schools. From a technological view we follow AAL technology trends by integrating a successful interface in a heterogeneous services AAL platform, focusing on a functional view. Educationally, a very flexible interface in line with communication training allows dynamic adjustment of complexity, enhanced by an accessible mindset and virtual elements significance already in use, offers specific interaction feedback, adapts to the evolving needs and capacities and improves the personal autonomy and self-confidence of children at school and home. TICO-home-control was installed during the last school year in the library of a special education school to study adaptations and training strategies to enhance the autonomy opportunities of its pupils. The methodology involved a case study and structured and semi-structured observations. Five children, considered unable to use commercial home control systems were trained obtaining good results in enabling them to use an open home control system. Moreover this AAL platform has proved efficient in training children in previous cognitive steps like virtual representation and cause-effect interaction.

## 1. Introduction

Access to highest possible autonomy and independence levels is a right every person has, as understood by our culture and reinforced by laws in different ways. In Spain its expression is an obligation for all society in general and the education community in particular.

Both an engineering school (University of Zaragoza, Spain) and a special education school (Alborada, Zaragoza, Spain) are engaged in a common long-term ongoing program to develop and try ICT technology solutions in Ambient Assisted Living (AAL) environments for training, education and personal autonomy support. From a technological perspective we look at it as an integrated service platform, bringing support for heterogeneous services in an open low cost ICT system based as much as possible on mainstream products. From the perspective of the education community, environmental control is a tool that comes to support the right to access the highest possible autonomy.

Bringing together both perspectives meant overcoming accessibility burdens by taking advantage of as much previous training in interaction as possible and make new training as widely useful as possible. It was easy to conclude that the communication interface already developed in collaboration with the special education school, TICO [1], should be the one every other training service in the AAL platform should be consistent with.

Many former works in the AAL field have developed and tested several platforms and a variety of standards looking both at technological challenges as middle-wares and integration of heterogeneous systems or new sensors or functions and at accessibility challenges for people with severe mechanical or cognitive disabilities, as shown in the state of the art review.

The technological system described in this paper has as its major goal the accessibility to meet functional specifications as collaboratively designed by different education community, university and special education school agents: to make available an open and low cost tool with enhanced accessibility to explore how far children can go in the use of home control and similar services with appropriate school training. Consequently the technology development focuses on accessibility, modularity, simplicity and preferentially off-the-shelf mainstream technologies and communications.

The university authors began this action after the experience of several European and national projects dedicated to smart homes and ambient intelligence in the AAL field, as described later. The long term idea is to explore the highest possible autonomy training by using this AAL platform, adapt it to children’s needs, bring the AAL platform with home control service to children’s home and further integrate new useful AAL services with the same mind-set and a similar interface.

Moreover, as TICO is a widely used communication panel editor, this strategy helps teachers already using TICO to easily prepare home-control user interface panels in a feasible and simple way, as they are already doing for communication purposes.

Inclusion of the interface they already use for many interaction and communication training scenarios was expected to give better results in both training directions: children with already some communication interface skills would more rapidly access the environmental control; on the other direction, training in interaction using home control as a feedback tool would also improve their communication skills.

We have come to a simple and modular system that can already be implemented based on third party commercial elements. It is installed in the library of Alborada, the special education school in which this work is performed.

The environmental control system uses a Raspberry Pi (RPI) board that by regular ICT communication standards is able to manage different proprietary and free systems, such as X10, Lonworks, and multimedia communication standards such as infrared and WiFi, among others.

### 1.1. Related Works

This action is framed in the context of AmI and AAL. It is related and will be integrated with Time Orientation Training for children in special education schools. Several long term actions have been consolidating the cooperation of the team involved in this action, special education teachers and university technological teachers, paying special attention to communication paradigms and actions supported by technology with the aim of supporting personal autonomy [2,3].

The Ambient Intelligence (AmI) vision described by Weiser [4] started the AmI paradigm which is inherent to the AAL paradigm. Specifically in AAL the need for integration of heterogeneous technologies in a seamless way and ease of use by a non-technological user have been identified [5].

AAL activity has faced the need to coordinate the knowledge and methodologies of different disciplines as human needs, human machine interactions, integration of heterogeneous technologies and middle-wares among others, for it is a field in which technology comes especially closer to human beings’ daily way of living, implying understanding of capacities, preferences, needs and applications and involving human sciences methodologies for education, training and evaluating the impact of a technology in the daily living and performance of a person.

Former projects of this research team from which experience is condensed into the presently described work are Ashored (1992), SCALP (1994), and some recent ones like AmiVital (2006), the MonAMi project (2010) and Siamyd (2011) [6].

Recent AAL work about interface design for AAL [7] describes a few current commercially available systems using pictograms for daily tasks, although focusing different aspects of the interface: ActivePal [8], that deals with the amount and contextualization of the information to be displayed; ADLife [9], in which they introduce a new model which is directed at supporting a holistic way of thinking about AAL systems considered as an integration of several useful services instead of a single commercially successful service.

Of course the need for adaptable and adaptive user interfaces in AAL has been addressed by previous works [10], building up models to extract relevant information from the user, environment (context) and interactions. Some works start from solutions for interfacing technologies and also come to enhancing personalization of interface (URC) [11], having a similar idea of using validated standards, although much closer to a technological view.

### 1.2. AAL Platforms and Meeting User’s Needs

AAL is promoting platforms which are able to integrate several services, each benefiting from the contextual and user information, and offering users a coherent frame for support. Most works include a combination of a technological way of thinking with a human interaction way of thinking, as we can see in the next related papers, although usually biased to a technological view as many of the promoters of actions in AAL are technology teams.

Software engineering has offered several works and technological development methodologies that take advantage of software structured development. One of those is Unified Modeling Language (UML). In [12] the authors describe the technological architecture and over it they manage to meet user needs, not showing any specific modulation of technology based on user interest or taking into account other disciplines. Another example is [13], in which UML is used for developing an interesting concept of famiWare, which gives a good description of contextual information and handling. They focus on technological challenges showing user needs as something already decided or put into value. This paper also shows how the use of UML is very adequate to this type of approaches, in which the focus is set on the technological structures and their functionalities.

A similar work is [14], which clearly states the functional goals to be met in the authors’ focus, giving good reasoning for the relevance of such topics and making them the starting point of their development. No methodology or process is shown or mentioned to integrate ideas and perspectives from different disciplines, nor to keep them aware through the project, which probably was done anyhow, may be in a non-structured way, for this work describes evaluation by several non-technological disciplines. Scenario methodology is implicitly shown and structured evaluation with users reported. Description of interaction among several disciplines involved is not shown, further than the evaluation process in which users are involved. Similarly, in [15] user needs are taken into account focusing them on the technological aspects and solutions although no methodology is shown in user needs nor evaluation.

Several other works include some referenced methodology for user requirements. A work in the health field with case-study methodology is described in [16], summarizing in two case studies all their goals (long term monitoring and smart hospital). Modular schemes describing service components add to component interactions that give an approximation of information flows, although it seems oriented to technological people, and not so much understandable for non-technological profiles in our particular multidisciplinary work experience.

Based on user needs and with the aim of improving alert handling, [17] does have a representation of functionality as an algorithm description, and contextual information is also displayed. It does have two case studies with which requirements are validated and their argumentation explanation is shown and applied. The degree of integration of the activities is very good.

Other software methodology used for AAL platforms is Service Oriented Architecture (SOA), as in [18], where authors provide a complete view of the experiences gained in the Middleware Platform for empowering cognitive disabled and elderly (MPOWER—IST 034707 project) with respect to using model-driven development (MDD), showing how techniques for SOA can be successfully applied to the AAL domain. The method used is to investigate and record the user needs, define a set of reusable software services based on these needs, and then implement pilot systems using these services.

A very interesting review centered on the Service Oriented Architecture (SOA) paradigm is the work of Alves et al. [19] which covers the use of methodologies and concludes that evidence for adoption of the methods is not mature, given the primary focus on model examples. The proposed approaches still have serious limitations in terms of rigor, credibility, and validity of their findings and most approaches still lack tool support addressing the heterogeneity and mostly textual nature of requirement formats.

A singular study to be highlighted in this regard is [20], which proposes the use of 2001 International Classification of Functioning, Disability and Health (ICF) taxonomy from the World Health Organization [21], concluding that AAL is too technology-based, even when stating that works are user-centered. This paper proposes an ICF-based taxonomy to evaluate contextual factors influencing efficiency and acceptance of AAL services, bringing technology further into its human interaction side.

Several AAL platforms have been developed aiming integration of heterogeneous technologies and services. Two of them have a large development in different projects, UNIVERSAAL [22,23,24], which promotes an open platform for AAL service delivery and users’ involvement, and OSGI4AMI (for example the MonAmi project [25,26,27]) [28]. Current work may benefit from any of such platforms, and can also benefit them by integrating a common communication path as we have done with TICO. Our work does not go further in interoperability, understood as the technical ability to communicate with different pieces of technology: it goes further in considering communication with the special education community. Of course it is done from a conscious understanding of importance of interoperability. In this reference study we show those other excellent developments with which our proposed perspective can be integrated in the future. The described action deals with interoperability in the technological interface in a much simpler way, having also solved it, and adds integration with communication tools in the human-machine interface.

Examples of use of such platforms and similar ones can be found in several AAL projects. SOPRANO integrates several services like reminders to take pills, and social activity [29,30,31]. PERSONA [32,33,34], Amigo [35], Oasis [36] among others are also examples of the use of such platforms. Another example is Accessibility and Usability Validation Framework for the AAL Interaction Design Process (VAALID) [37,38,39] which focuses on developing an integrated development environment for computer-aided design and validation of user-interaction subsystems that improve and optimize the accessibility features of AAL services, aiming for social inclusion and independent living of senior citizens. It is done with a 3D-immersive simulation platform to facilitate the user-interaction system design.

As a final conclusion of our state of the art survey we can say, following Mavrommati [40], that the research community in AAL has been centering its attention in what is technologically feasible (and meritable) [41,42,43], biasing the work consequently towards lacking enough attention to personalized interfaces to meet and adapt to user’s needs and preferences.

## 2. Experimental Section: Materials and Methods

The described work implements an AAL platform which includes environmental control and TICO as the communication interface. It also has other AAL services, such as Time Orientation Training service, surveillance and communication services, although those will be described in subsequent papers, centering attention here on using TICO as a global interface for home control.

The described home control system is installed in the library of the Alborada Special Education School. Any teacher acquainted with TICO can elaborate the interface panels for a specific child, with no need of TICO development team intervention.

From all the available population in pupils at Alborada, seven children were identified as unable to use a commercial home control and considered with possibilities to use TICO. From those seven, finally five children underwent the training hereby described, with the aim of improving their autonomy and trying TICO as facilitating interface for AAL services.

### 2.1. Global Collaboration Project Phases (this Paper Includes the First 4) and Goals

*Project phases*:

1. Adaptation of the AAL platform: on the field requirements as far as interface, functionality, simplification for each expected type of pupils.
2. Installation and trial.
3. User selection.
4. User training and conclusions: further adaptations needed, useful learning strategies.
5. Home installation of useful modules.
6. Manuals and guidelines, further improvement, updates, system and training dissemination.

*Goals*:

- Improving autonomy of people with special needs and consequently, their quality of life.
- Improving self-esteem and motivation for interaction, based on individual capacities and autonomy.
- Promote and assist task realization by people with special needs.
- Achieve access and control to daily-use-technological elements: lighting, TV, shutters, music systems...in a safe and comfortable way.
- Measure the autonomy degree achieved by users and measure user satisfaction levels that can be observed.
- Take advantage of feedback in the real world of virtual elements to help the learning process of cause-effect interactions by checking relationships among virtual elements and their activation consequences.

### 2.2. Description of the Technological Tool

#### 2.2.1. Requirements from the Education Community

The described project resulted from a confluence between experience in AAL service development in the university team and a study of the potential for improving personal autonomy in the special education team:

The interaction has to be “TICO-like”, or best based in TICO as interface. They wanted children to be trained with electrical devices (control over plugs), a TV set, music equipment, motorized shutters and lights as ways to improve the personal autonomy of children.

A requirement present from the beginning was to meet standards and regulations to be able to bring the AAL platform into the homes of the children, so the learning will impact directly their personal autonomy. Reliability and overall maturity of the system is a prerequisite for installation and training.

Even though the education community didn’t impose any requirement about the system to be used, cost accessibility is always a consideration in technology for this field. To be able to adapt the platform to different situations and preferences, like new buildings and already inhabited homes, we chose to integrate different home-control standards, proving also the capacity of integration of heterogeneous systems, choosing commercial modules when possible. Of course the home control needs to be configurable, flexible and reliable.

#### 2.2.2. AAL Platform and TICO-Home-Control Description

Besides the cost reduction obtained with mainstream technology and the immediate availability of the necessary technology, we found it more practical to communicate with different standard modules than to build them ourselves, so we can rely on the manufacturer compliance with European legislation and speed up the installation, so we have integrated several standards that can be managed from the same TICO interface, making the standard they use or their manufacturer transparent to the user. It integrates several standards to allow selection of the domotic system to adapt to the user: X10, KNX, ZigBee, 433 MHz, infrared and communication standards, such as WiFi and RS232 or RS485.

We understand standards will continue to progress and we are not trying to make a contribution to this respect, only to the way they are integrated with the interface so children gain accessibility and can be trained. If a new standard is found to take the lead in the future, we could maintain the operative system and transparency to the user by integrating communication with it.

Domotic systems on the market have been previously considered by the team as an option for training and evaluated as reliable, composed by a large number of elements, expensive or very expensive, especially those which offer some kind of flexibility or configurability. Most are closed systems, modifications cannot be made with the version the final user buys, so integration with TICO had to be done through an external platform that integrates those commercial systems.

**Figure 1:**
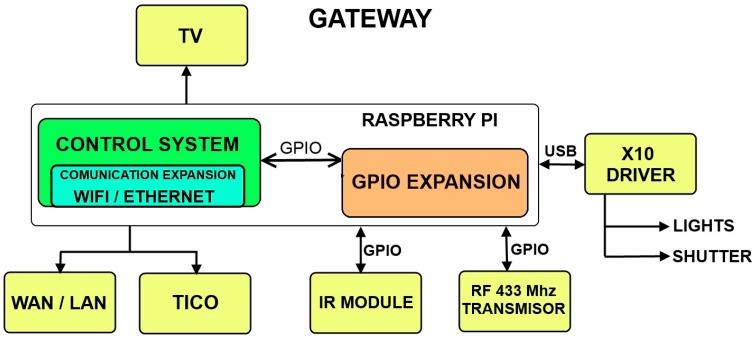
Modular description of AAL platform in Alborada.

Consequently, this AAL platform basically consists of a centralized computer that communicates with every other module of the system and focuses specifically in user interaction. Figure 1 shows the modular description of AAL platform installed in Alborada.

**Figure 2:**
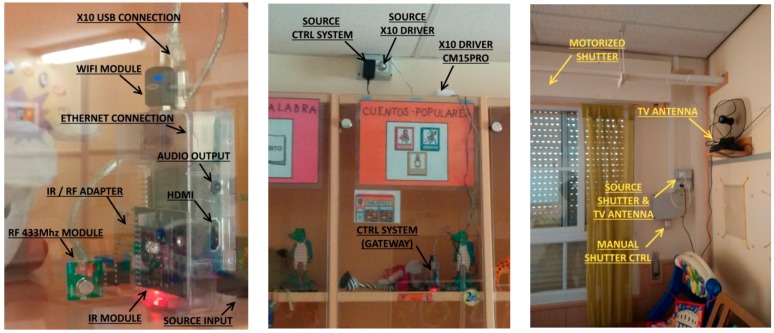
AAL platform based in RPI and various modules.

**Figure 3:**
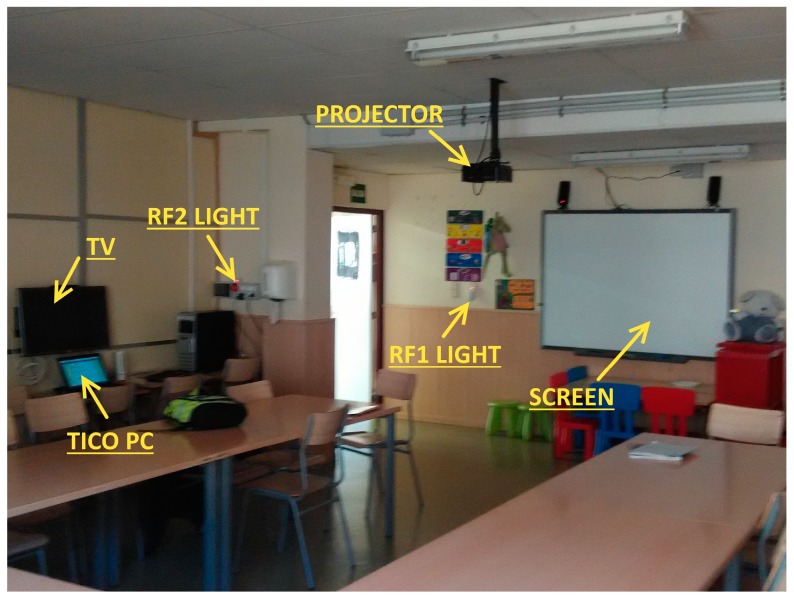
Several actuators and elements in the library.

**Figure 4:**
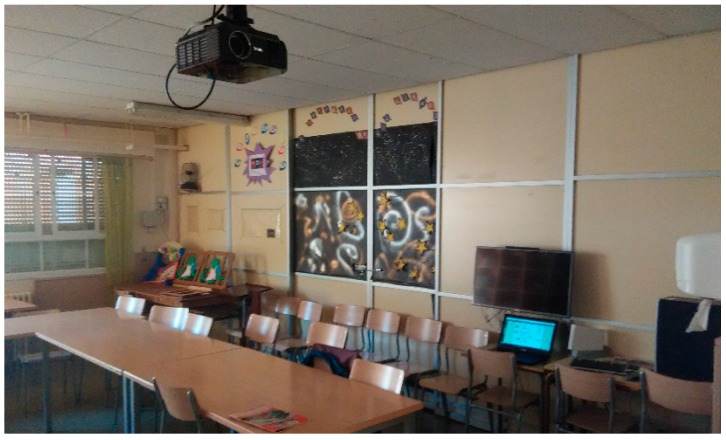
Shutter control and library overview.

**Figure 5:**
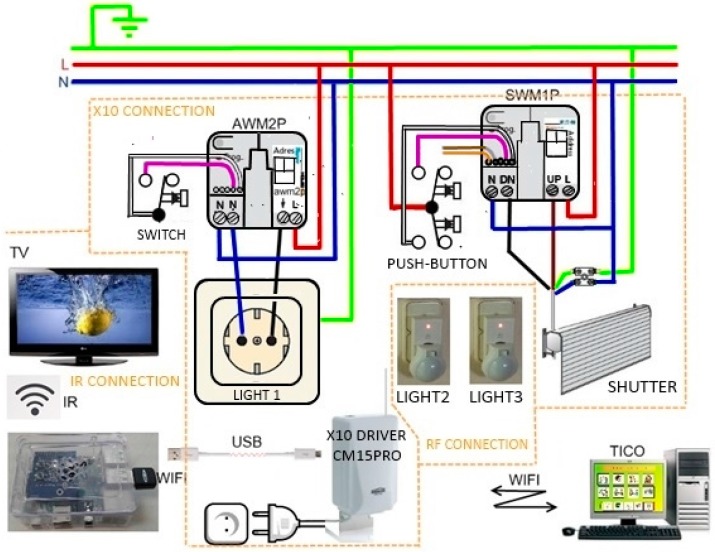
Electric diagram of the Alborada installation.

The platform is composed off a RPI module connected to Marmitek X10 plug module and shutter motor modules through their CM15Pro bridge, both through power line and RF communications. TV IR control signals are emulated by means of a PiCobber IR Remote. We have studied connection through RF 433 MHz with systems as CHACON, EURO BRIC, LIFE DOM, REMOTE CONTROL SOCKET SET. Figure 2, Figure 3 and Figure 4 are images of the installation used for training in Alborada school library. Figure 5 shows the logic and functional diagram of the AAL installation.

**Figure 6:**
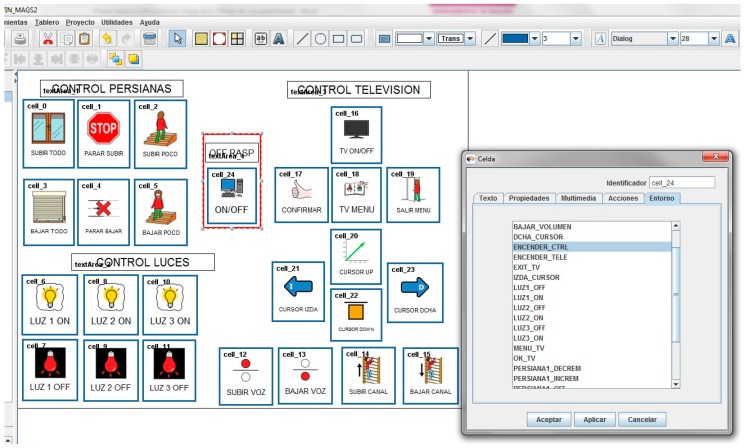
TICO-home-control interface.

Standard TICO cells were modified by the TICO development team to send a preconfigured message when activated that our platform receives and understands, being able in this way to make home control panels operate in the same easy way as the communication panels edition, as Figure 6 is showing. For more information about TICO readers may refer to [1].

**Figure 7:**
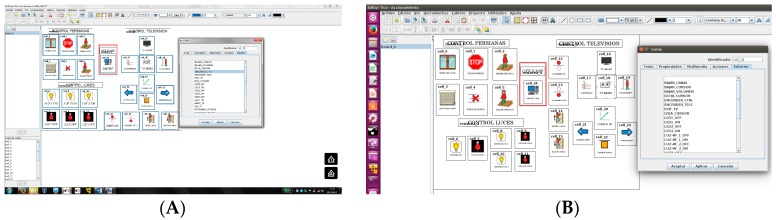
(**A**)TICO-Home-Control in windows; (**B**) TICO-Home-Control in Linux.

**Figure 8:**
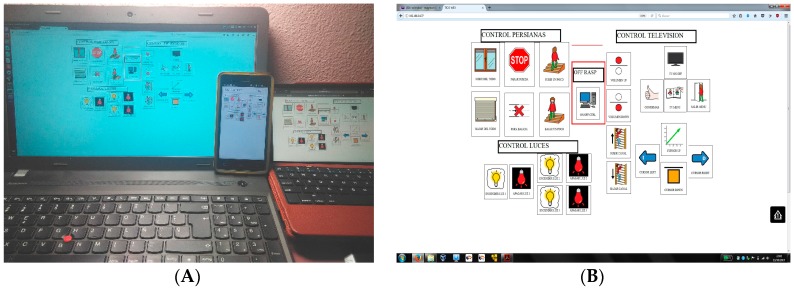
(**A**)TICO Home Control in several platforms; (**B**) TICO-Home-Control in web page.

Home control will have many elements and they can be activated from different panels, so each user may have a personalized panel that includes only the elements he/she can manage or the ones found of interest. For this reason, dynamic adaptation to the performance of the trainee during training sessions is simple and allows for training to low-frustration-capacity children. It also allows training with a simplified system and the progressive addition of more elements until reasonable complexity for the user is reached. In this way, it is expected that daily use of the system may allow for further expansion, as easy to edit the panel as teachers do daily for communication training purposes. Figure 7 and Figure 8 show TICO-Home-Control running in different OS and hardware platforms.

**Figure 9:**
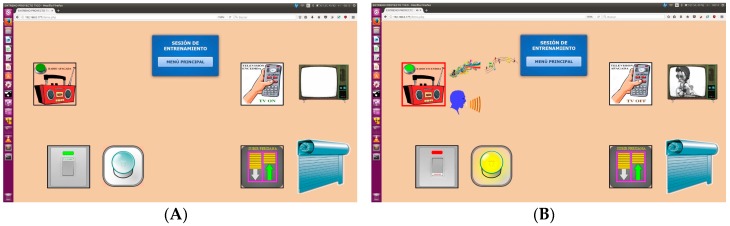
(**A**)Main window for training session and (**B**) Actions in training session.

Simplified panels like those used for training are shown in Figure 9, with different representations when a device is activated.

## 3. Results, Experimental Section

This section describes the experimentation and results of training five children selected from among the pupils of Alborada Special Education School, in TICO-Home-Control, as example of AAL platform which uses an established communication tool to facilitate human interaction and display panel elaboration. It shows how TICO makes it easy to adapt the interface to different stages of training allowing stages with very simple cognitive requirement and gradually increasing complexity to provide with more complete functionality. It highlights the appearance of a somehow common strategy that can be further used for new children to be trained. It shows how those children can use a home control system, improving their personal autonomy. It also shows there is an impact in some basic interaction training as relating virtual representations with real objects or functions and cause-effect interaction basics. Finally, it shows an impact on the satisfaction and attitude of children, that is included here as we understand it is a crucial element in learning concepts and skills. We understand this work offers a contribution to generic AAL platforms by showing a way of improving their usability and accessibility both through gradual training and permanent communication interface.

### 3.1. Methodology

The target population is special education children that were formerly considered unable to use a standard home control but considered able to learn it with TICO incorporation. Users show various disability types and in various degrees, so methodologies based in control and experimentation groups are not feasible, as the groups would be too scarce in number and too heterogeneous in capacities to provide any statistical significance. Consequently a case studies methodology is used, making a comparison in the skills observed before and after the training intervention and registering the incidences, results and observations of each session. The intervention is structured in the following phases:

(1) Meeting with the education community to jointly present the project (both school and university).
(2) Criteria definition for user selection (motor disability, cognitive level and maturity level).
(3) For each selected user, there is a joint study by the research team with the school physiotherapist team about the optimum access way to the system interface.
(4) Meeting with selected-user counselors to inform them about the training process and goals.
(5) Training in identification of virtual and real elements in the training room, focusing on the relation between real and virtual elements and the understanding of the function triggered in the real world when a virtual cell is activated.
(6) Training in a simplified four cell TICO home control panel (TV, window, music, siren) as previous work to increasingly complex panels or complete TICO panel, using division of elements in working panels as an intermediate or definitive simplification for TICO home control usage.
(7) When capacity is sufficiently proven in previous phases, training on a complete TICO panel (nested windows with full functionality for each house element).
(8) Improvement of the user interface, simplified or complete TICO panel and any other modifications considered useful for installation of such system at the user’s home, considering also furniture adaptations and cognitive simplifications.
(9) Overall training conclusions, attending also to former described goals of motivation, self-esteem, autonomy improvement and associated satisfaction, management capacity rating and aspects to improve after the training process.

Training sessions were individual and lasted 30 min each. The training place is the library of the school, where the AAL platform is installed. Initial planning of each session is standard for all users, so the same instructions and similar steps are given to each user which allows comparison of access difficulties.

#### Users Selection and Management

Seven subjects between 7 and 21 years old were pre-selected, from both “Compulsory Basic Education” (CBE) and “Adult Life Transition” (ALT) groups. After prior evaluation of the adequacy of users, five out of the initial seven were selected for training, considering the other two were in too immature a development state.

Children were selected after studying their cognitive and physical interaction capacities. All of them were selected among those considered as not able to use a regular commercial home control systems and expected to be able to respond positively to this specific progressive training and to benefit in their personal autonomy from having a home control system at home. Four children are in CBE level and 1 adult in ALT.

**Figure 10:**
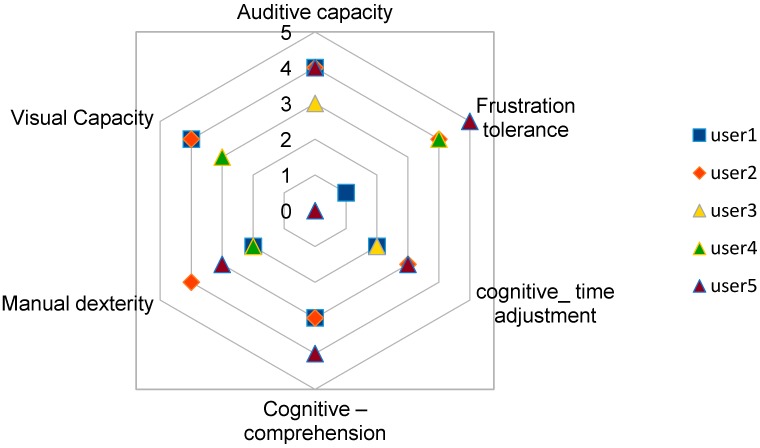
Relevant capacities of selected users for managing TICO interface.

Some specific capacities were considered as most relevant to be able to use TICO interface and follow corresponding training: auditory and visual capacities, manual dexterity, cognitive capacity to understand, to adjust to timings and tolerance to frustration. Their capacities were graded from 0 (null capacity) to 5 (normal capacity), being subjectively assessed by an experienced special education teacher, having the values shown in Table 1 and illustrated in Figure 10.

### 3.2. User1, 8 to 10 Years Old, CBE Level, Girl

**Figure 11:**
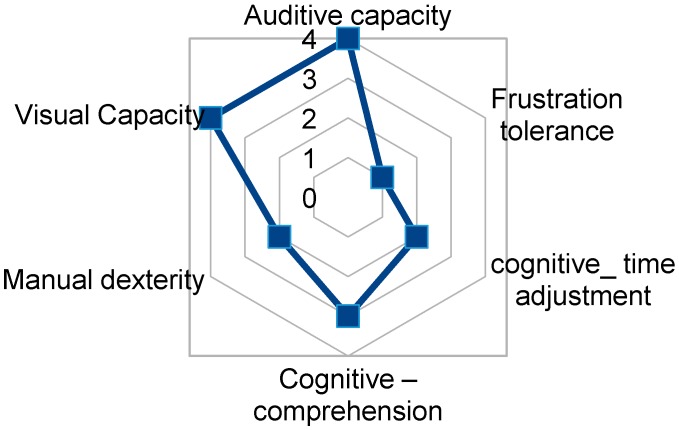
TICO interface use relevant capacities fingerprint for User1.

User1 has a good cognitive level and manipulation skill that allows her, after training, to access to TICO panel for environment control. User1 can use the mouse with her left hand, needing support to help with waiting times or sweep-selection software. Her capacities are shown in Figure 11. Considering her initial evaluation by the physiotherapist team, she has the possibility to use directly a tablet as a physical interface as her motor dexterity allows her to point to the screen elements without difficulty.

#### 3.2.1.1st and 2ndSessions: Introduction to the Interface

Both first sessions are dedicated to getting acquainted with the interface. the 1st goal for User1 is to recognize the elements of the training room that are represented in the interface (TV, shutters, light, music equipment, …). She progresses well and doesn’t show any difficulty in recognition of the correspondent elements in the real world.

Once completed the first training step, we pass to observe her reaction and management capacity with the four cell TICO panel (television, window, music, siren). She identifies all cells and she is able to move and place roughly the mouse in the desired cell or the one she is asked to, although she needs to improve her control.

#### 3.2.2. 3rd Session

Her fine psychomotricity is checked up as she is asked to work with the tablet. She shows capacity to pulse and manage the screen options quite fluently, although both these aspects are still object of training.

She is offered two sizes of tablet to work with TICO panel: one has a 10” screen and the other a 7” one. She performs better with the second, so that is the one chosen for her training.

In this session User1 is presented the complete TICO--Home-Control interface panel. To facilitate more specific observation of the elements that form the panel and her training, the screen is divided in three areas of work: shutter, lights, TV.

User1 recognizes most pictograms that represent real elements in the room and shows more difficulty with those that imply more abstraction, such as functions like "stop" to stop the movement of the shutters or a cross to stop any action.

As far as locating pictograms she shows good skill, she is able to locate them and verbalize their function when each cell is pointed to her: moving up or down the shutters, controlling each light, turning on or off the TV set.

Globally the training is positive and User1 shows great satisfaction when she activates the cell “move shutter up” and checks it does so, or any other action, move it down, turn on any light or the TV.

As far as conclusions relating to the later personalization of the system for installation at her home, it is concluded that panel, as shown in Figure 6, Figure 7, Figure 8 and Figure 9, should be simplified in following aspects:

- Shutter control: Leave cells “shutter up”, “shutter down” and “stop” (that stops both movements).
- Light control: Only two cells: “turn on” and “turn off”.
- TV control: Leave only three cells. One to turn it on/off; another two cells to increase or decrease the channel selection.

Training process assessment is positive: for User1 there have been several moments of satisfaction when checking she is able to produce actions in an independent way to turn on the TV, move the shutters, turn off the light, which are actions of daily life for which she normally needs support from another person.

Moreover training has given her the opportunity to include basic skills, as adjust to waiting times, improvement her fine psychomotricity, new vocabulary acquisition, lateralization and spatial orientation.

### 3.3. User2, 8 to 10 Years Old, CBE Level, Boy

**Figure 12:**
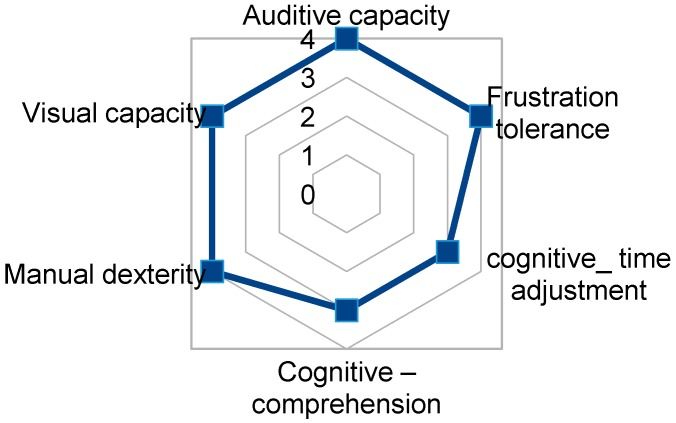
TICO interface use relevant capacities fingerprint for user2.

User2 presents good cognitive level and manipulative dexterity that allows him access and functioning of TICO panel for home control with some training (Figure 12). This user also shows the ability to use the mouse with his left hand as access medium and also show ability to point at with this hand. From a previous study he is considered capable of using a tablet for training, being necessary to evaluate his accessibility and the appropriate size for his capacities.

#### 3.3.1. 1st & 2nd Sessions 

Both first sessions are dedicated to getting acquainted with the interface. First we considered the recognition in the room of the elements that will be object of the training work in the interface (TV, shutters, lights, music equipment). He doesn’t show difficulties to identify and establish the relation between the real object and virtual representation as a pictogram.

After this first phase we pass onto the observation of his reactions and capacity in management of TICO panel with four cells. He identifies cells and elements without major difficulty. He is able to use the mouse to stop in the cell he wants or he is asked to, although there is a need to improve control. Time in sweeping mode to highlight one cell and then another needed to be increased.

#### 3.3.2. 3rd Session

**Figure 13:**
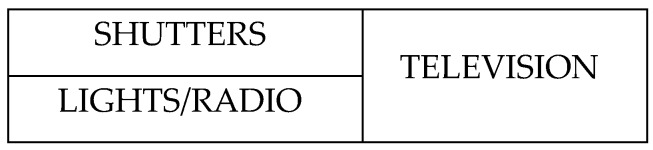
Grouping of areas in interface panel for simplified training.

Fine psychomotricity is observed when working with the tablet. He is able to pulse, point and manage with some fluency. Accessibility improves for User2 with the tablet compared to the regular PC and the mouse. Two sizes of tablets are tried for TICO panel, with 10” and 7” screen dimensions, showing better results with 10” screen, so training will be made with this size of tablet. When exposed to complete TICO panel for environmental control, the screen is divided in three working areas: shutters, lights and TV, as shown in Figure 13.

User2 recognizes all pictograms representing real elements in the room, and has difficulties with those that don’t correspond with a real object and are more abstract (stop, cross to stop a cell activation, move up/down shutter a little bit), as already found in User1.

After training he is able to show understanding of the meaning and the function of each element. He is able to identify each cell that corresponds with the desired pictogram and verbalizes the function each performs. Also he is able to point at the location of each pictogram to move up or down the shutters, turn lights on or off and the same for the TV. Even so, it is convenient for User2 to decrease the number of cells and simplify their content.

Training has been positive. User2 has shown great joy when observing the activation of the real elements when he activates the corresponding cells, especially when turning on the TV and selection of channel until reaching one he likes.

Regarding the later installation at his home there is a need to simplify the panel, proposing an initial reduction to two parts of the screen instead of three: shutter control and TV, then, progressively including more elements in the panel with lights or other white-goods.

a) Shutter control: decrease cell number to three: move up, move down and stop.
b) TV control: again only three cells, one to turn on/off, and two arrows to select channels.

Assessment of the interface training is positive: for User2 it has been very satisfactory to check he was able to actuate elements in the room in an independent way and only by pointing at the pictogram, being elements of everyday actions like turning on the TV, moving the shutters or turning the light off.

As also happened with the rest of users, the training has also worked on some previous skills, like adjustment to waiting times, improvement of fine psychomotricity, new vocabulary acquisition, lateralization and spatial orientation.

### 3.4. User3, 11 to 15 Years Old, CBE Level, Girl

**Figure 14:**
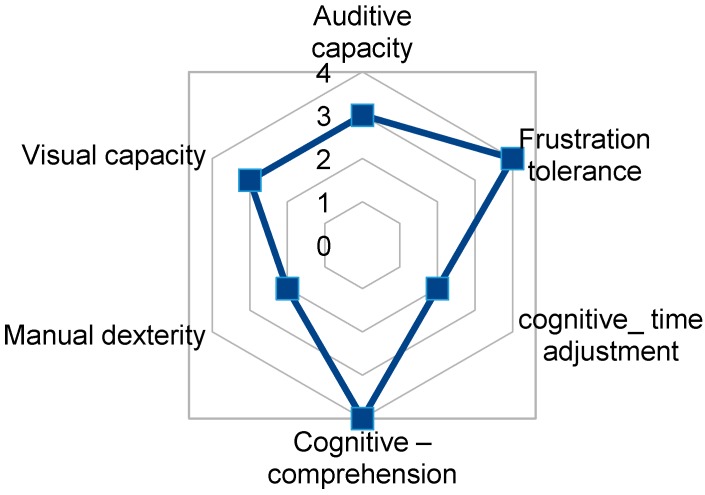
TICO interface use relevant capacities fingerprint for user3.

User3 shows an enough cognitive level and manipulative dexterity that allows her to be a candidate for training in home-control TICO (Figure 14). She is able to use the mouse with both her left and right hands, mainly with left one. She has a tremor that interferes very much with her controlled movements, so it is necessary to keep in mind that her waiting times will be longer.

Once verified her mobility she has with the mouse and also her ability to point on a tablet screen, it is concluded that it is easier for her the accessibility with the tablet. Then we had her use two sizes of screens, which shows as a result that she adjusted better to the smaller (7”) one. 

She is able to activate panel cells in the tablet, improving her precision with the smaller one, as her tremor minimizes with palmar attachment. She also improves using her left hand fingers, although she alternates with both left and right.

#### 3.4.1. 1st & 2nd Sessions 

Both first sessions are dedicated to getting acquainted with the interface. We first identify the elements to train with in the room, television, shutters, lights, music equipment. She doesn’t show difficulties identifying and establishing the relationship among real objects and pictograms.

Afterwards, we observe her reactions and her capacity to use a four cell TICO panel (TV, window, music, siren). She identifies all four cells and their correlation with pictograms. TICO is being configured to make automatic sweep with a 5 s delay between one cell and following one, hoping she can have enough time to select the pictogram and pulse to activate it.

Her tremor makes it also difficult for her to activate her selected cell, so the delay time is further increased. Validation of her performance is positive. Consequently training is moved to the next phase to work with a complete TICO panel.

#### 3.4.2. 3rd Session

After the result with the training of her previous mates she is presented a TICO home panel divided in three parts as with User2, so she can acquire domain over each of them, as shown in Figure 13. She recognizes pictograms in each cell and the function they perform. She feels very motivated and calm. She locates the pictograms when asked for, pointing at correct cell and activating real function. In this session training focuses on shutter control and lights control.

#### 3.4.3. 4th Session

Focus of training is set on the TV control for this session, progressing later with training of the complete TICO home control panel. Regarding subsequent installation at her home, simplification of TICO home control panel is suggested:

a) Shutter control: leave three cells, shutter up, down and stop.
b) Light and plug control: only two cells, one to turn on and another one beside it to turn it off.
c) Television control: Only three cells, one to turn on/off, and other two to search for channel upward and downward. Volume could be suppressed to gain further simplification.

The training process has been satisfactory for this user, as she finds she could perform by her own common daily actions without other person’s support.

### 3.5. User4, 16 to 21 Years Old, CBELevel, Girl

**Figure 15:**
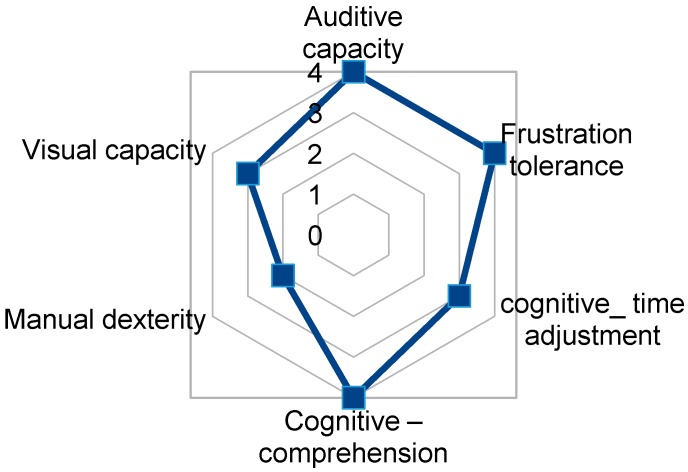
TICO interface use relevant capacities fingerprint for user4.

She shows a good cognitive level and enough manual dexterity, so she is considered candidate for TICO home control (Figure 15). She is an electric wheelchair user and navigates autonomously. She can use the mouse when she is adequately positioned on her chair.

Bearing in mind her capacities and competence level, and following her physiotherapist recommendations, training starts directly with 10” screen tablet, as she presents motor capacities to use furniture adaptations as needed.

#### 3.5.1. 1st Session

The first session is dedicated to getting acquainted with the interface. As with former users, training starts by recognition of the virtual and real elements that will appear in the interface (television, shutters, lights, music equipment). User4 has no difficulties in identifying and establishing the relationship among real objects and their pictograms.

Having in mind her positioning on the wheelchair, a lectern is placed on her table attached to the wheelchair, so she can have good access to her tablet. Goodness of fit of this adaptation will be validated through the training.

TICO home control is presented to User4, identifying all but one pictograms, she asks about the one for which she doesn’t know the associated action.

The complete TICO panel is divided in two parts to focus on the elements of each, in a similar way as shown in Figure 13. It is necessary to provide her with longer required time until she points to the cell and activates it. When she does, she communicates her joy to observe she can directly move up the shutter and that this way she could do it on her own at home. She has been able to activate all cells corresponding to shutter control and light control without difficulties.

#### 3.5.2. 2nd Session

Training is continued with the second part of the panel as divided in former session, which corresponds to the TV. It is observed that even when she is able to activate each cell of TICO panel having her tablet in a lectern attached to her wheelchair, she could better perform with further adaptation consisting in an articulated arm to support the tablet at the same height as her chest and at an adequate distance for her to access in a comfortable way.

It is also observed, regarding her home installation, that tablet cells should be enlarged in size to achieve better accessibility for her, as her visual field is affected. Then it is proposed to have a starting point with few cells and then have a gradual increase in number of cells, with the expectation that she will achieve better performance and capacity for an increasingly larger number of cells. Another good starting point considered could be to have the cells divided in two related panels with which she could train in all cells without suppressing any and then progressively offer her an integrated and complete TICO home control as her use at home would be evolving.

### 3.6. User5, 16 to 21 Years Old, Blind, ALT Level, Girl

**Figure 16:**
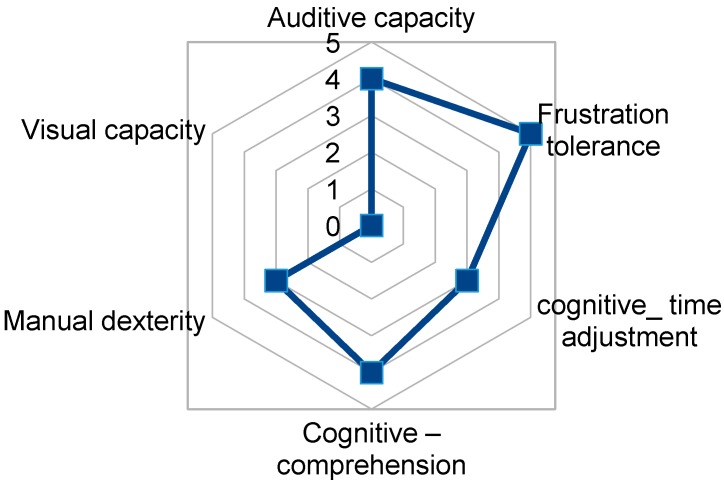
TICO interface use relevant capacities fingerprint for user5.

User5 has both motor disability and total blindness. Her cognitive level and manipulative dexterity is expected to allow her to use home control TICO with previous training (Figure 16). She can use the mouse with enough time in the automatic sweep with voice interface and sound feedback. 

TICO is prepared to have the swept cell related word spoken out loud so it also has accessibility with no visual feedback.

#### 3.6.1. 1st and 2nd Sessions

Both first sessions are dedicated to getting acquainted with the interface. The explanation of the elements that are objects of the four cell TICO panel and complete TICO panel are given to her orally. She knows what the selected elements in the training room are for, as well as how that would be at her home.

In order to evaluate her reaction and coordination capacities when choosing the moment to pulse to select a cell after she has heard its description, in a first stage only a four cell TICO panel is used (television, window, music equipment and siren). It is proven that she is able to listen with attention to the voice describing each cell in the sweeping process and to verbalize the function of each of the elements.

She shows very good precision in the sequence “listen to sweep and pulsed desired cell”. Anyhow and due to TICO design, it is necessary that the active cell be converted into full screen mode to facilitate the selection. She is also able to check by audio feedback the effect of each cell selection.

#### 3.6.2. 3rd Session

The complete TICO-Home-Control panel is not adapted to auditory function yet, so we use the support of an adult to emulate this function and she was trained in the activation of each cell of complete TICO home control panel, perceiving the auditory feedback of moving the shutters or turning the TV on. In order for complete TICO home control panel to be autonomously accessible for this user, several modifications are needed:

1. Simplify the TICO panel in two parts: for training this is done with TV control and music equipment control.
2. TV control simplification: she likes to listen to the TV programs and follow TV series even not seeing the images. She needs only three cells, turn on/off and two others to raise and lower the channel selection. Here again volume could be dropped in order to further simplify the panel, at least in a first stage.
3. Music equipment control: she loves listening to music. The panel would consist of two cells, one to turn on/off and a second one to select tracks from a CD.
4. Include reading function in the complete TICO home control panel.
5. Establish an adequate time delay in sweep function to adapt to her reaction time to listen and chose the desired function.

## 4. Discussion

All five cases have been given positive results in the TICO home control training, acquiring enough skills to be able to operate it at least in a simplified version, and gaining enough understanding of the functions associated to each virtual cell to find benefit for their personal autonomy. Results are summarized in Table 2.

Joint study of their previewed capacities with physiotherapists team have resulted in finding accessibility enough for TICO managing and several proposals of furniture adaptation, panel simplifications, and configuration of delay times that are found to be progressively improved with training.

Identification of virtual and real elements in the training room and their functional relationship has been achieved with all users, finding special difficulty with pictograms not directly representing real objects but abstract functions instead (as stop for shutter movement) mainly by the youngest children, users 1 and 2.

All users have performed satisfactorily with the simplified four cell TICO home control panel which has also been useful to further study the best available access mode and interfacing hardware platform for each one of them.

Training on a complete TICO panel has been performed gradually starting from a simplification by dividing elements in groups, two or three, finding good expectation of progress towards fully operability in several cases.

Based on observation, personalized proposals for improvement of user interface regarding home installation are described, which vary from simplifications of the number and size of cells, further adjustment of waiting times, grouping of cells in function-coherent areas in the interface, furniture adaptations and auditory feedback inclusion.

In all cases training has proven useful to acquire basic operational skills, as cause-effect training, relation among virtual and real elements, improvement of motor skills and comprehension of expected triggered functions, vocabulary, lateralization and spatial orientation.

As far as satisfaction with learning, mood and motivation, there is an overall autonomy improvement and a clear associated satisfaction, which expresses also as happiness and self-esteem. Observation of progress with training also concludes that management capacity is likely to improve by further autonomous training through usage, so personalization should be revised after weeks of installation and use at home.

## 5. Conclusions and Future Work

A simplified AAL integrated service platform has been developed to demonstrate the usefulness of integration with a successful interface in special education schools. It has been done following principles of integration of heterogeneous systems, modularity, interoperability among services, low cost and flexible configuration.

As AAL services, we can consider two services are integrated to prove the adequateness of their interaction, on one side a special education field recognized alternative and augmentative communication program (TICO) which provides for enhanced accessibility and coherent cognitive mind-set with all other communication efforts and trainings done with target population. The second service is home control integrating heterogeneous technologies, following the requested functions by special education teachers and based on the expected improvement in autonomy that it may offer.

The target population at the first stage is children that up to now were considered unable to use a home control system. The goal is to widen it to all population of special education children for whom home control may mean an improvement in personal autonomy. The target goes further beyond reported training trials to have it installed in the homes of those children for use in everyday life.

Selection of users for training is performed following the above principles and in cooperation with a physiotherapist team, who assessed the best accessibility option for each user that later is checked with direct experimentation.

Training was a success in all five cases, considering there were two additional children that were preselected and then discarded from this training phase due to higher difficulties. The team considered it best to deal with them when more experience has been gained in the progression of training for higher cognitive difficulties.

Installation of a TICO home control was recommended in all five cases with several modification proposals, most of which can be done with current system by configuring panels and parameters such as delay times in sweeping function, size and number of cells, grouping of cells, and various hardware platform for interface among others.

As far as self-esteem, mood and attitude towards the new system the training has been a success, rising solid expectations of improved autonomy. Besides training in usage of the TICO Home Control system, this platform has been found useful to train in basic cognitive and operational skills: Especially significant are training in time management about waiting and reaction times, training in cause-effect relationship with different observable feedbacks, lateralization and spatial orientation improvement and improvement of fine psychomotricity, as well as establishing relationships among virtual and real elements.

### Future Actions

Further training is to be performed with several disability types and severity degrees with children in the Alborada Special Education School. For the most skilled ones the aim is to improve autonomy by using home control services. Regarding the less skilled ones, the aim is to use AAL platform as training for previous operational and cognitive skills as seen in the article.

Installation in the homes of the children who are found with capacity to use it will be a further step. Then the impact on life quality of the children and family will be evaluated too. Once there are a significant number of installations, another study will be performed for finding relations among the capacities of children and configurations achieved, with the aim of providing with some guidance to teachers and families in this regard and elaborate educational material for special education schools.

New services are to be integrated also in this AAL platform to further check the benefits of this specific interface as far as accessibility and usability are concerned and to offer a more complete technological AAL suite to the educational community. Implementation of supervision and alarm raising service and time management service and training tools is under progress.

## Figures and Tables

**Table 1:** Interface-relevant capacities of selected users.

User Capacities	User1	User2	User3	User4	User5
Auditory capacity	4	4	3	4	4
Visual capacity	4	4	3	3	0
Manual dexterity	2	4	2	2	3
Cognitive—comprehension	3	3	4	4	4
Cognitive-time coordination	2	3	2	3	3
Frustration tolerance	1	4	4	4	5

**Table 2:** Summary of experimental results.

			USER 1			USER 2			USER 3				USER 4		USER 5		
USER PROFILE	AGE range		8 to 10			8 to 10			11 to 15				16 to 21		16 to 21		
	PREVIOUS CAPACITY STUDY	COGNITIVE LEVEL	OK			OK			OK				OK		VERY GOOD		
		MANUAL DEXTERITY	OK			OK			OK, tremor				OK/in wheelchair		VERY GOOD		
	ALTERNATIVE DEVICE		TABLET 7”/MOUSE LEFT HAND			TABLET 10”/MOUSE LEFT HAND			TABLET 7”/MOUSE BOTH HANDS/improves with palmar attachment				TABLET 10”/LECTERN attachment to wheelchair		TOTAL BLIND MOUSE to select, AUDITIVE FEEDBACK		
TRAINING SESSIONS	NUMBER OF SESSION		1	2	3	1	2	3	1	2	3	4	1	2	1	2	3
	SESSION GOAL		Recognition of room elements		COMPLETE TICO PANEL	Recognition of room elements		COMPLETE TICO PANEL	Recognition of room elements Introduction to TICO interface		COMPLETE TICO PANEL, division in 3 areas: shutters and lights/ TV		Recognition of room elements	TV ELEMENTS OF PANEL	Recognition of room elements		COMPLETE TICO PANEL
	OBSERVATIONS		Good reactions, rough control movement		Improves psychomotricity, movement precision, overall management	Good reactions, Good relation virtual and real elements		Fine motricity improves with 10” tablet	No difficulties in recognition of all pictograms, Good managing skills		Very motivated, calm, improves precision, difficulty with tremor		No difficulties in recognition of all pictograms Happy with exercises	Visual and motoric difficulties Needs larger cells	No difficulties in recognition of all pictograms Coordinates time between auditive feedback in sweeping software and action to select		Performs successfully with emulated auditive feedback support
	POSITIVE RESULTS		Identifies cells correctly, improves mouse control		Locates all elements recognizes most pictograms Verbalizes expected cell function	Relates pictograms with real world elements and actions Good use of mouse.		Locates all elements and cells, recognizes all pictograms representing real objects, verbalizes expected cell function, Can manage by his own	No difficulties in recognition of all pictograms Good managing skills		Easily locates requested cells, Improves motricity by training		No difficulties in recognition of all pictograms Very happy with success	Manages TV successfully	Acquires precision in listening and activating cells No cognitive difficulties		Good coordination among listening and activating actions
	DIFFICULTIES		Rough control with mouse		Identification of function for abstract pictograms	Needs longer delay times in sweep functions		Abstract pictograms meaning	Tremor in hands Needs longer waiting times for sweeping		Tremor in hands		Needs longer delay times in sweep functions	Needs improvement in location of tablet, furniture adaptation	Total blindness Motoric difficulty		Needs temporarily auditive feedback emulation as it is not fully included yet
EVALUATION	TRAINING		positive			positive			positive				Positive		positive		
	OPERATIONAL SKILLS		positive			positive			positive				Positive		positive		
	SATISFACTION OBTAINED		positive			positive			positive (motivated and calm)				positive and happy		positive (and happy)		
	AUTONOMY IMPROVEMENT		Fully autonomous with simplified interface			Fully autonomous with simplified interface			Fully autonomous with simplified interface				Fully autonomous with simplified interface		Fully autonomous with final previewed interface		
	OTHER SKILLS TRAINED		Adjust waiting times, fine psychomotricity, new vocabulary acquisition, laterality improvement, Spatial orientation improvement			Adjust waiting times, fine psychomotricity, new vocabulary acquisition, laterality improvement, Spatial orientation improvement			Adjust waiting times, fine psychomotricity, new vocabulary acquisition, laterality improvement, Spatial orientation improvement				Adjust waiting times, fine psychomotricity, new vocabulary acquisition, laterality improvement, Spatial orientation improvement				
PROPOSED ADAPTATION	INTERFACE SCREEN		reduce number of pictograms			reduce number of pictograms			Needs simplification				Needs simplification		Needs simplification		
	SHUTTER		3 buttons: up/down/stop			3 buttons: up/down/stop			3 buttons: up/down/stop				3 buttons: up/down/stop				
	LIGHTS		2 buttons: on/off						2 buttons: on/off				2 buttons: on/off		MUSIC: 2 buttons: turn on/off, select track		
	TELEVISION		3 buttons: on/off, up channel, down channel			3 buttons: on/off, up channel, down channel			3 buttons: on/off, up channel, down channel				3 buttons: on/off, up channel, down channel		3 buttons: on/off, up channel, down channel		
	OTHER		Overall satisfaction			Overall satisfaction			Very motivated and calmConscious of autonomy improvement potential at home				Proposed to start with simplified panel. Expectations of progressing to complete panel by improvement by usage		Needs larger cell sizeNeeds full auditive feedbackNeeds configuration of waiting times in sweep function		
